# Unveiling the pathogenic mechanisms of *Clostridium perfringens* toxins and virulence factors

**DOI:** 10.1080/22221751.2024.2341968

**Published:** 2024-04-09

**Authors:** Anny Camargo, Juan David Ramírez, Raymond Kiu, Lindsay J. Hall, Marina Muñoz

**Affiliations:** a Centro de Investigaciones en Microbiología y Biotecnología-UR (CIMBIUR), Facultad de Ciencias Naturales, Universidad del Rosario, Bogotá, Colombia; b Health Sciences Faculty, Universidad de Boyacá, Tunja, Colombia; c Molecular Microbiology Laboratory, Department of Pathology, Molecular and Cell-Based Medicine, Icahn School of Medicine at Mount Sinai, New York, NY, USA; d Institute of Microbiology and Infection, College of Medical and Dental Sciences, University of Birmingham, Birmingham, UK; e Gut Microbes and Health, Quadram Institute Bioscience, Norwich Research Park, Norwich, UK; f Norwich Medical School, University of East Anglia, Norwich, UK; g Instituto de Biotecnología-UN (IBUN), Universidad Nacional de Colombia, Bogotá, Colombia

**Keywords:** *Clostridium perfringens;* toxin type, genetic diversity, host, enteritis, toxinotypes

## Abstract

*Clostridium perfringens* causes multiple diseases in humans and animals. Its pathogenic effect is supported by a broad and heterogeneous arsenal of toxins and other virulence factors associated with a specific host tropism. Molecular approaches have indicated that most *C. perfringens* toxins produce membrane pores, leading to osmotic cell disruption and apoptosis. However, identifying mechanisms involved in cell tropism and selective toxicity effects should be studied more. The differential presence and polymorphisms of toxin-encoding genes and genes encoding other virulence factors suggest that molecular mechanisms might exist associated with host preference, receptor binding, and impact on the host; however, this information has not been reviewed in detail. Therefore, this review aims to clarify the current state of knowledge on the structural features and mechanisms of action of the major toxins and virulence factors of *C. perfringens and* discuss the impact of genetic diversity of toxinotypes in tropism for several hosts.

## Introduction

*Clostridium perfringens* is a Gram-positive anaerobic bacterium that can form spores that are crucial during transmission. *C. perfringens* spores are exceptionally resistant to stressful environments, such as high temperatures, the presence of oxygen, or low nutrient levels [1]. These features facilitate its survival in different environmental niches, including soil, faeces, sewage, food, and the intestinal tract of humans and animals [2]. This “survivability” means that *C. perfringens* has been associated with many infections and diseases, including being the second leading cause of foodborne bacterial disease in the United States (causing one million illnesses yearly) and Europe's fourth leading cause [3–5]. In England, 8-13% of foodborne gastrointestinal outbreaks are estimated to be associated with this bacterium (90,000 cases of *C. perfringens* per year) [6]*.*

The impact of *C. perfringens* infections is due in part to the bacterium’s potential to secrete multiple extracellular toxins, including alpha-toxin (CPA, *cpa/plc* gene), beta-toxin (CPB, *cpb* gene), epsilon-toxin (ETX, *etx* gene), iota-toxin (ITX, *iap,* and *ibp* binary genes), *C. perfringens* enterotoxin (CPE, *cpe* gene) and Necrotic enteritis B-like toxin (*NetB*). These toxins correspond to essential toxin genes used in the current toxinotyping scheme (toxinotypes A-G) (Figure supplementary 1) [7].

Each toxinotype is associated with specific diseases. For example, toxinotype A, characterized by the presence of the *cpa*^+^ toxin genes, is linked to gas gangrene in humans and animals. Toxinotype B carries the *cpa^+^*, *cpb^+,^* and *etx*^+^ genes, and it is associated with haemorrhagic enteritis in calves, foals, and sheep, as well as dysentery in lambs. Toxinotype C, with the toxin genes, *cpa^+^*, *cpb^+^,* and *cpe^+/-^*, is linked to enterotoxaemia in sheep and necrotizing enteritis in humans (pigbel), pigs, calves, goats, and foals. Toxinotype D, featuring *cpa*^+^, *etx*^+,^ and *cpe*^+/-^, is associated with enterotoxaemia in lambs (pulpy kidney disease), goats, and cattle. Toxinotype E, containing *cpa*^+^, *itx*^+,^ and *cpe*^+/-^ genes, is linked to enterotoxaemia in calves and lambs. Toxinotype F, with *cpa^+^* and *cpe^+^*, is associated with food poisoning and antibiotic-associated diarrhoea (AAD). Finally, toxinotype G, presenting *cpa^+^* and *netB^+^*, is associated with avian necrotic enteritis [7].

Additionally, *C. perfringens* produces other clinically relevant accessory toxins, such as Perfringolysin O or theta toxin (PFO) and *C. perfringens* beta 2 toxin (CPB2). Although not used for toxinotyping, these toxins can act synergistically with extracellular toxins, impacting other toxins’ expression, production levels, and virulence factors, thereby influencing overall disease progression [8].

The diversity of clostridial toxins and other virulence factors remains a critical study point. Therefore, in this review, we present a brief updated description of the structural and molecular characteristics, mechanisms of action, and genetic diversity of the main toxins and virulence factors associated with the tropism of *C. perfringens* toxinotypes across a diversity of hosts, including links to specific disease phenotypes.

## *C. perfringens* toxins: biological properties, function, host tropism and diversity

### Localization of toxins of *C. perfringens* and plasmids families

The virulence of *C. perfringens* largely depends on its ability to produce toxins, which can be encoded on plasmids, chromosomally, or both. So far, seven groups of plasmids have been described for *C. perfringens*: transfer of clostridial plasmids (Tcp), pCP13 *C. perfringens* (Pcp), pIP404, phage-like, small plasmids or an unclassified group [9], and recently the botulinum conjugation in *C. perfringens* (Bcp) group of plasmids encoding a new putative conjugation locus.

The first significant group of plasmids carries the Tcp conjugation locus and genes encoding clinically relevant toxins such as *cpb2* and *cpe*, iota binary toxin genes *iap* and *ibp*, as well as antibiotic resistance genes, including chloramphenicol, clindamycin, erythromycin, bacitracin, and lincomycin [10, 11]. Although a *C. perfringens* strain can carry up to five similar plasmids, it was recently demonstrated that the stability and inheritance of these plasmids are favoured by the ResP recombinase that catalyses the multimer resolution system [12] and by differences in the type II partitioning systems (ParMRC) [13].

A second major group of *C. perfringens* conjugative plasmids is the pCP13-like plasmid family, which shares a highly conserved sequence at the Pcp locus [14, 15]. These plasmids carry a *cpb2* consensus variant associated with disease in horses and piglets [16]. They also have the novel C. perfringens binary enterotoxin (BEC/CPILE) [14], detected in the faeces of gastroenteritis patients, food [17], and healthy UK children [18].

The PiP404 plasmid family consists of small non-conjugative plasmids that encode a bacteriocin; however, they have not been identified to carry any toxin genes [19]. The newly identified Bcp plasmids contain a novel putative conjugation locus (Bcp) with a sequence like *Clostridium botulinum* plasmids and contigs that have the same plasmid mobilization gene, mobC and the same zeta toxin-encoding gene as pJGS1984_5. Additionally, these plasmids encode VirB4 homologous proteins and VirD4-like conjugation proteins distinct from the variants encoded by the Tcp and Pcp plasmids [20].

The presence of toxin-carrying plasmids and antibiotic-resistance genes in *C. perfringens* has been associated with its survival ability in multiple environments. Moreover, the high rate of plasmid transfer in this bacterium (2.9 × 10^−1^ to 3.8 × 10^−2^) also allows it to overcome fitness costs and segregation loss. This influences the transformation of toxinotypes in a given environment, especially within the gastrointestinal tract, implying impacts on disease outcomes [21].

However, in some cases, the metabolic load and additional energy resources required by plasmid replication, disruption of essential host genes by plasmid gene integration, and plasmid-encoded molecules may negatively impact native host proteins, leading to decreased fitness in bacterial cells [21, 22].

In addition to plasmid-localized toxins, other toxins genes such as *cpa* (also *plc*) and *pfo* are located only on chromosomes [9]. Furthermore, *cpe* can be located on the chromosome, as shown in food poisoning isolates or on a large plasmid, as observed in non-foodborne gastrointestinal disease and veterinary isolates.

The location of toxin-associated genes is expected to influence disease outcomes, as plasmid-encoded toxins can be transferred by conjugation to strains of *C. perfringens* toxinotype A that reside as part of the “normal” intestinal microbiota, giving rise to new strains with enhanced colonization and virulence traits. Conversely, chromosomal variants may be lost over evolutionary time due to mutation or deletion events and may cause shorter disease durations due to low colonization levels and spread [21].

## Toxin structure

*C. perfringens* toxins are diverse and widely distributed. Structurally, clostridial toxins may have similar binding domains but different catalytic domains, suggesting possible recombination in toxin evolution. This recombination could occur through horizontal plasmid transfer or insertion-deletion processes, serving as adaptation, survival, and tropism mechanisms. This recombination could occur through horizontal plasmid transfer or insertion-deletion processes, serving as adaptation, survival, and tropism mechanisms [23]. As outlined below, some toxins are composed of different domains. In addition to allowing flexibility and dynamics in the toxin, these domains promote and facilitate the recognition of specific substrates in the target cell.

## Major toxins of *C. perfringens*

**Figure 1.**
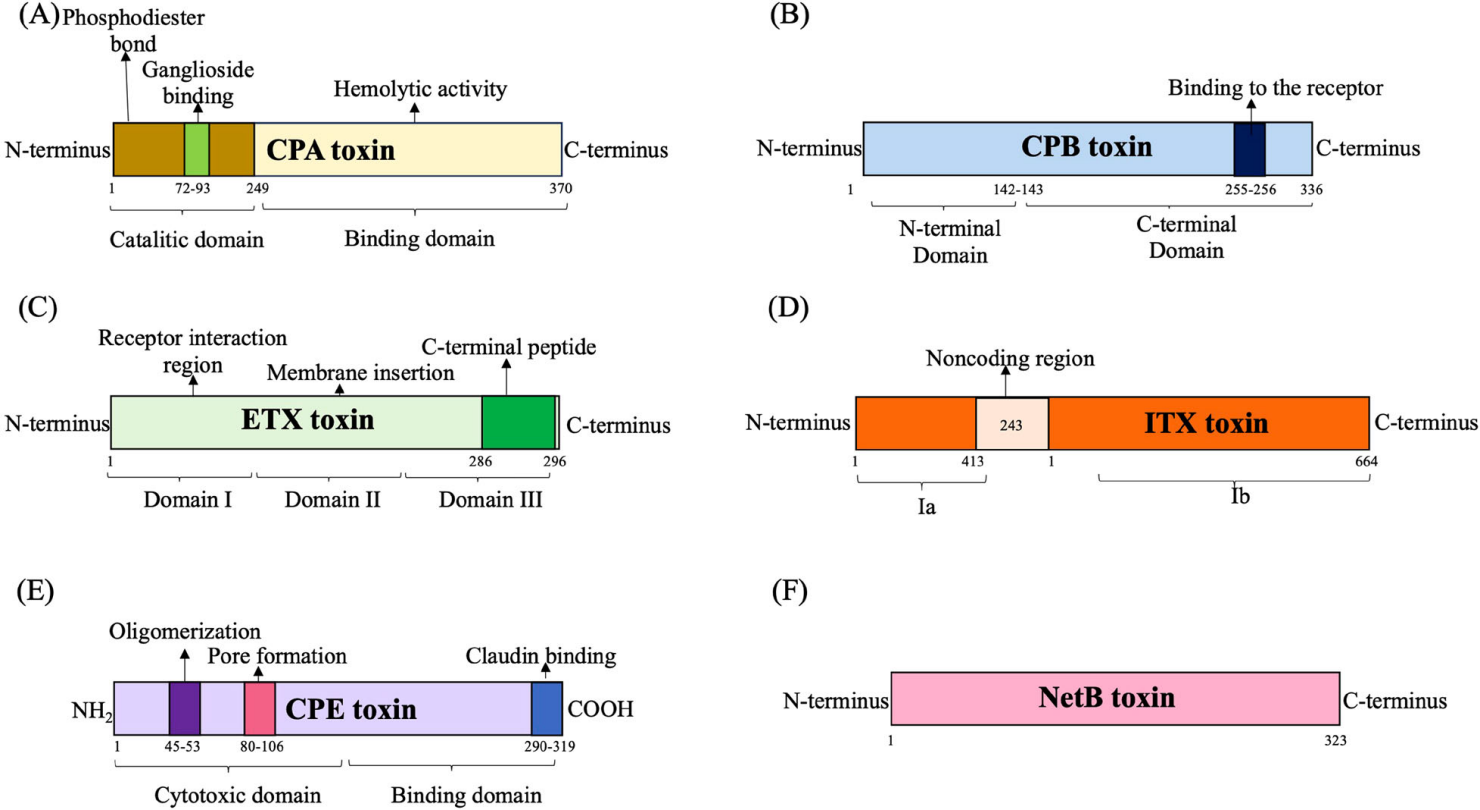
Schematic representation showing domain structure of *Clostridium pefringens* main toxins (A). CPA toxin (B). CPB toxin (C). ETX toxin (D). ITX toxin (E). CPE toxin and (F). NetB toxin are shown. Numbers indicate amino acids that mark domain boundaries.

CPA is composed of 370 amino acids (aa) and is divided into two domains: the catalytic N-domain (CP1-249) and the membrane-binding C-terminal domain (CP247-370) [24] (Figure 1a). It involves cell membrane colonization, haemolytic activity, and damaging action [24–27].

CPB toxin, a 336 aa protein (Figure 1b) in the alpha-haemolysing family, is suggested to bind to its receptor or form the oligomer via its C-terminus (although the exact structure–function relationship is still under study) [28].

ETX toxin is composed of three domains: i) the amino-terminal domain, involved in the receptor binding process; ii) the central region domain, responsible for membrane insertion plus channel formation; and iii) the carboxy-terminal domain, involved in the activation of proteolysis [29] (Figure 1c).

ITX is a binary toxin composed of two components: an enzymatic Ia (454 amino acids) and a binding Ib (875 amino acids), separated by 243 non-coding nucleotides. Both components, Ia and Ib, exhibit cytotoxic properties [30] (Figure 1d).

CPE is a single-chain polypeptide consisting of 319 amino acids and three domains: I, II, and III. Domain I binds to the claudin-specific receptor, while domains II and III constitute the N-terminal region associated with pore-forming activity [31] (Figure 1e).

NetB, a putative toxin gene encoding a 323 aa protein (Figure 1f), including a 30 aa secretion signal sequence, which has similarity to CPB toxin (38% identity), leading to its naming as necrotic enteritis β-like toxin, NetB [32].

## Another clinically relevant accessory toxins

PFO has 500 aa residues and a 27-residue signal peptide. It is composed of four domains, with domain four (D4) mediating the toxin's binding to the eukaryotic cell's plasma membrane [33].

The structure and receptor of CPB2 remain unclear. It is suggested that only a small fraction of amino acid segments adopt an α-helix conformation in both beta toxins (atypical and consensus), which is insufficient to cross a membrane. However, in the case of consensus CPB2, some amino acid segments have been identified that could form transmembrane β-chains [34].

## Mechanisms of action of *C. perfringens* toxins

*C. perfringens* is a pathogen of significant clinical and veterinary importance, attributed to its capacity to induce disease in various hosts by producing different toxins (Table 1. Section A, B).

**Table 1.** Classification of toxins and other virulence factors of C. perfringens.

Virulence factor or toxin	Gene	Biological Properties and Function	Associated disease	Reference
**Section A. Toxins used for *C. perfringens* typing**				
alpha-toxin	*plc, cpa*	- Modifies cell membranes by enzymatic activity. - Zinc-dependent phospholipase C which degrades phosphatidylcholine and sphingomyelin, in the eukaryotic cell membranes.	- Gas gangrene. - Necrotic enteritis in chickens. - Enteritis in calves and in piglets. - Sudden infant death syndrome (SIDS). - Inflammatory diseases.	[35]
*Clostridium perfringens* enterotoxin	*cpe*	- Intestinal damage, severe villus shortening, along with epithelial necrosis and desquamation.	- Human foodborne illness - Antibiotic-associated diarrhoea - Human enteritis necroticans - Sporadic diarrhoea - Enteric diseases in swine, cattle, horses, sheep and goats and wild animals such as deer and bears.	[36]
Alpha-clostripain	*ccp*	- Processing of secreted proteins - Potential to affect the levels of active extracellular toxins.	- Possible Clostridial myonecrosis-associated	[37]
Epsilon toxin	*etx*	- Aerolysin-like b-pore-forming toxin family. - Intestine: Epithelial cell detachment and cell necrosis, lamina propria haemorrhaging and polymorphonucleocyte infiltration. - Affect renal system, brain, cardiorespiratory system, and pleura. - Hemolysin. - Blood pressure elevation, increased contractility of smooth muscle, vascular permeability increase.	- Enterotoxaemia in domestic ruminants (sheep and goats) - Illness in humans related to the epsilon toxin?	[38]
Beta toxin	*cpb*	- Beta-barrel pore-forming toxin family and forms oligomeric pores in several susceptible immune cell lines. - Vascular necrosis and marked inflammatory reactions result from a direct interaction of CPB with vascular endothelial cells. - Destruction of jejunal and ileal villous tip epithelium.	- Necrotizing enteritis (NE) in pigs, sheep, goats, calves, and humans.	[39]
Iota toxin	*itx*	- Necrosis of the superficial epithelium with relative sparing of the crypt epithelium, and submucosal haemorrhage and transmural haemorrhage.	- Lethal necrotizing - Enteritis and sudden death in beef calves.	[40]
**Section B. Accessory toxins**				
*Clostridium Perfringens* beta 2 toxin	*cpb2*	- Haemorrhage and necrosis of the small and large intestines. - Degenerated and necrotic desquamated epithelial cells, cell debris, inflammatory cells.	- Enteric diseases in swine, cattle, horses, sheep and goats and wild animals such as deer and bears.	[41]
Perfringolysin O	*pfoA*	- Tissue destruction and an anti-inflammatory response. - Vascular accumulation of leukocytes within blood vessels and the extracellular matrix of host tissues. - Disruption of endothelial, local edema and systemic shock and multiorgan failure.	- Myonecrosis (gas gangrene), haemorrhagic enteritis in calves and, septicaemia (intravascular haemolysis) in humans. - Associated with Necrotizing Enterocolitis in human neonates.	[11]
Necrotic enteritis E-like toxin	*netE*	- Pore-forming toxin within the Leukocidin/Hemolysin superfamily.		[42]
Necrotic enteritis F-like toxin	*netF*	- Putative beta-pore-forming toxin. - Pore-forming toxin within the Leukocidin/Hemolysin superfamily.	- Canine haemorrhagic gastroenteritis. - Foal necrotizing enteritis.	[43]
Necrotic enteritis G-like toxin	*netG*	- Putative beta-pore-forming toxin. - Cytotonic effects, such as proinflammatory effects.	- No confirmed association with disease.	[42]
Necrotic enteritis B-like toxin	*netB*	- Pore-forming toxin.	- Necrotic enteritis of chickens.	[44]
Toxin *C. perfringens* large cytotoxin	*TpeL*	- Ras-specific glucosyltransferase activity inactivating the Ras signalling pathway leading to apoptosis. - Cytotoxic effects (morphological changes such as enlargement and the rounding of Vero cells).	- Associated with avian necrotic enteritis.	[45]
Collagenase	*ColA*	- Degradation of the extracellular matrix due to their ability to digest native collagen.	- Associated with avian necrotic enteritis.	[46]
Binary enterotoxin of *C. perfringens*	*becA,becB*	- Enterotoxic activity. - Fluid accumulation in mice.	- Acute gastroenteritis in humans.	[17]
**Neuramidases**				
Secreted neuramidase	*nanJ*	- Increase CPE-induced cytotoxicity and CH-1 pore formation in Caco-2 cells.	- Contribute to food poisoning caused by F-type c-cpe strains carrying the *nanH* and *nanJ* genes.	[47]
Secreted major neuramidase	*nanI*	- Synergy with CPA toxin, ETX toxin, CPB-toxin and CPE toxin - Sialidase activity. - Promotes the colonization of *C. perfringens* in the intestinal tract and enhances the cytotoxic activity.	- Gas gangrene?	[47]
Non-secreted neuramidase	*nanH*	- Enhance CPE cytotoxicity. - Reducing host cell surface charge repulsion during CPE binding or removing sialic acid residues that sterically interfere with CPE binding.	- Intestinal pathology.	[48]
**Hyaluronidases**				
Hyaluronidases	*nagI, nagJ, nagH, nagK nagL*	- Facilitates the spread of the major tissue-damaging α-toxin, thereby potentiating its cytolytic activity. - Degrade hyaluronate cell surface coatings. - Act on connective tissue during gas gangrene. - Increased permeability of the connective tissues.	- Gas gangrene	[49, 50]

## Sphingomyelin and phosphatidylcholine degradation

**Figure 2.**
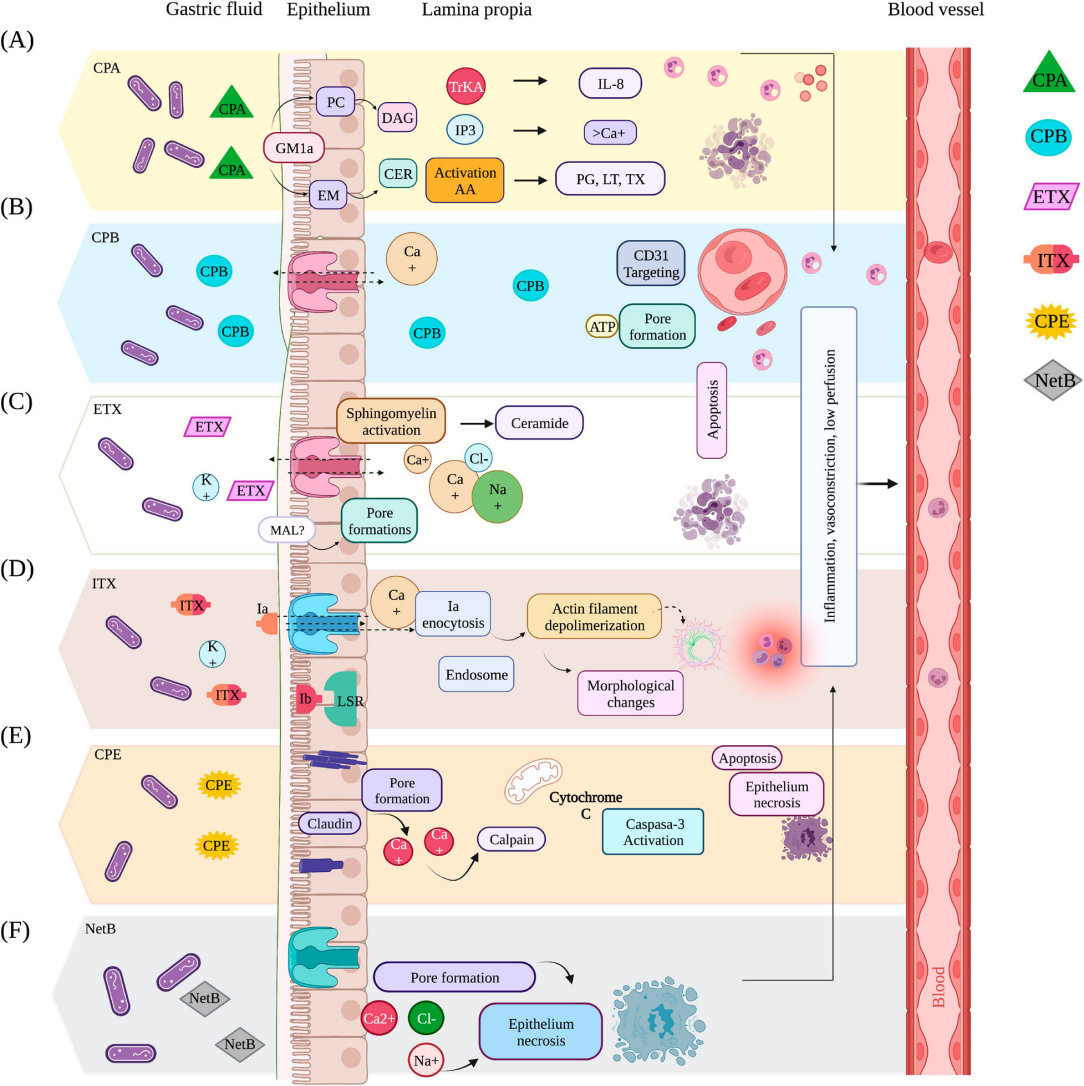
Action mechanisms of the primary *C. perfringens* toxins used for toxin typing*.* Molecular mechanism of action of the major toxins of *C. perfringens.* (A). CPA toxin: CPA toxin interacts with GM1a, hydrolysing phosphatidylcholine (PC) and sphingomyelin (SM), resulting in the formation of diacylglycerol (DAG) and ceramide (CER) with Tropomyosin kinase A receptor (TrKA) activation and triggers the activation of an intracellular signalling cascade with Interleukin - 8 (IL-8) release. The activation of phosphatidyl inositol 3 (IP3) promotes intracytoplasmic calcium (Ca^+^) entry (B). CPB toxin: CPB binds to platelet endothelial cell adhesion molecule-1 (PECAM-1) with subsequent release of adenosine triphosphate (ATP) and formation of pores that allow ion exchange to and from the cell (C). ETX toxin: ETX toxin interacts with protein “myelin and lymphocytes” (MAL), forming an active pore that induces ion transport and exchange across the cell membrane (D). ITX toxin: The binding of Ib to the lipolysis-stimulated lipoprotein receptor (LSR) receptor mediates its entry into the host cell, promoting the formation of channels for the entry of Ia by endocytosis with subsequent depolymerization of actin filaments, generating morphological changes and alteration of cell permeability (E). CPE toxin: the CPE toxin binds to claudin receptors, contributing to the formation of a pore on the cell surface with ion exchange and osmotic imbalance. (F). NetB toxin recognizes cholesterol-free regions in cell membranes by forming heptameric hydrophilic pores that allow the entry of ions such as Na^+^, Cl^-^ y, and Ca 2^+^.

**Figure 3.**
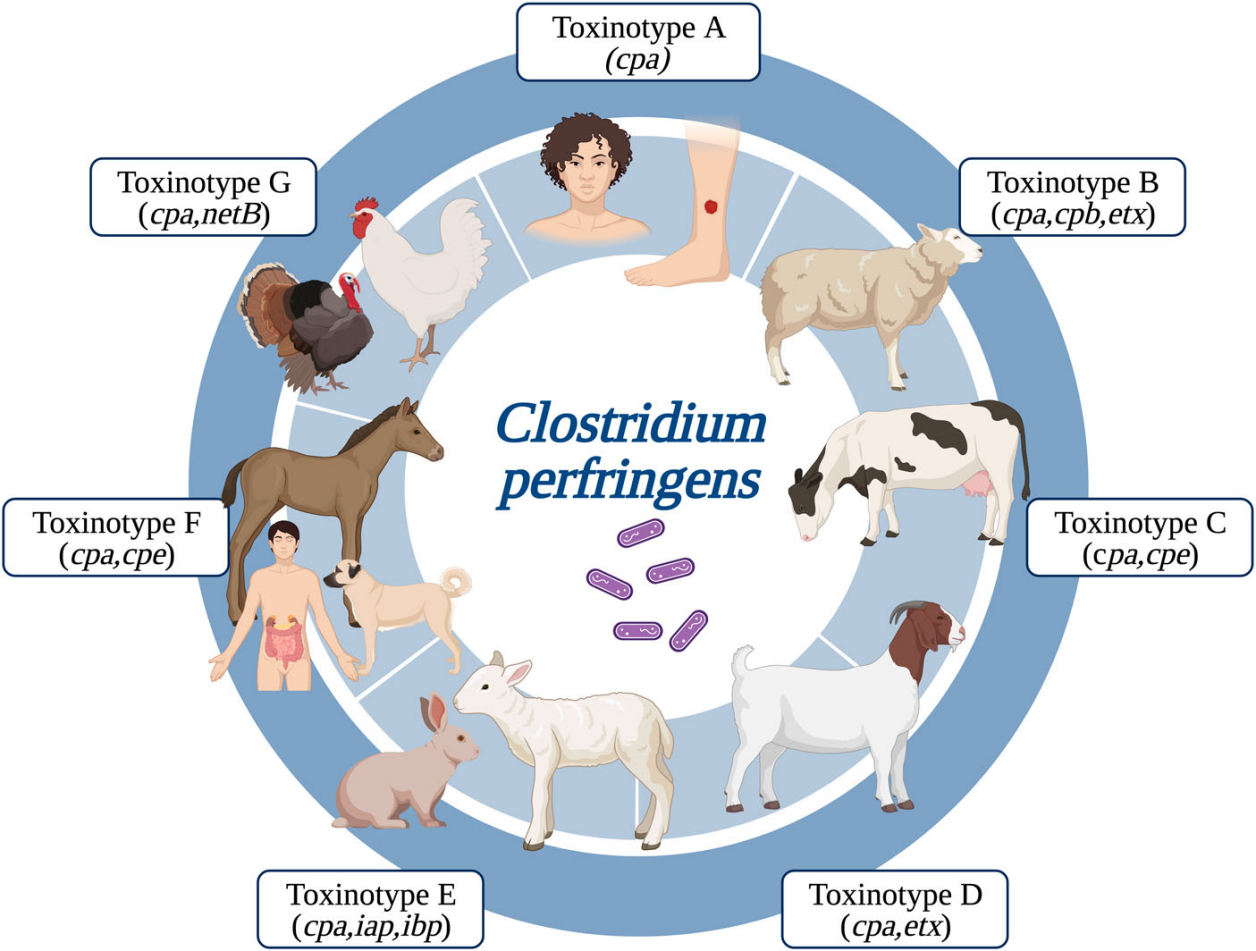
Schematic representation of principal hosts for each *C. perfringens* toxinotypes – each box representing a different toxinotype.

CPA, a toxin in all toxinotypes, is the primary determinant of virulence in clostridial myonecrosis caused by toxinotype A. The process is initiated by the toxin binding to the ganglioside receptor GM1a [51] (Figure 2a). This binding results in the degradation of sphingomyelin and phosphatidylcholine in the cell's plasma membrane, triggering proinflammatory responses during the early phases of gas gangrene [52]. This inflammatory process may advance to aggravated tissue infection, accompanied by secondary gas crepitation in the tissue and the development of necrotic in humans [35] (Figure 3).

## Formation of pores

Pore-forming toxins (CPB, ETX, ITX, CPE, and NetB) allow Ca2+, Na+, Cl- entry and K + loss with subsequent cellular electrochemical gradient depolarization, ionic homeostasis alteration, and cell death [43].

CPB is the primary virulence determinant in necrotizing human enteritis and enterotoxaemia caused by toxinotype C in sheep. It is also involved in haemorrhagic dysentery in sheep caused by toxinotype B (Figure 3). CPB binds with platelet endothelial cell adhesion molecule 1 (PECAM-1, also known as CD31) to trigger the formation of pores in the endothelial cell membrane. This process causes damage to the endothelial cells that make up the vasculature of the intestinal mucosa (Figure 2b), resulting in haemorrhagic and necrotizing enteritis [53, 54].

The ETX toxin, found in toxinotypes B and D, is linked to enterotoxaemia in ruminants (Figure 3). It binds to receptors dependent on cholesterol and sphingomyelin present on the outer membranes of endothelial cells lining the intestine and vascular cells in organs such as the brain, kidneys, and liver in ruminants. While the exact identity of the receptor is unknown, it is confirmed that ETX forms active pores, causing direct damage to intestinal endothelial cells. This process increases vascular and intestinal wall permeability, leading to intestinal epithelial cell detachment and necrosis, triggering enterotoxaemia, especially in goats (Figure 2c) [38, 55].

For ITX, present only in toxinotype E and associated with haemorrhagic enteritis in cattle (Figure 3), it is described that the lipolysis-stimulated lipoprotein receptor (LSR) mediates the entry of toxin Ib into the host cell [56], allowing the formation of functional channels for ion movement and entry of Ia by endocytosis [57]. This interaction leads to degenerative changes in the mucosal epithelium of the small intestine, increased permeability of intestinal cell monolayers, and haemorrhagic lesions in the serosa and mucosa characteristic of enteritis (Figure 2d) [58].

The CPE toxin, found in toxinotype F, is recognized as the primary toxin causing food poisoning and gastrointestinal illnesses in humans, including AAD (Figure 3). Its mechanism of action involves binding to claudin receptors, crucial components of the tight junctions between epithelial or endothelial cells at the cell surface. This binding facilitates the formation of pores in the plasma membrane [59], removing claudins from the cell membrane and disrupting tight junctions between cells. Consequently, this disruption increases barrier permeability in the small intestine and colon, impairing absorption and diarrhoea (Figure 2e) [60, 61].

NetB, found in toxinotype G of *C. perfringens* and associated with avian necrotic enteritis (Figure 3), recognizes cholesterol-free regions in the membrane of intestinal epithelial cells. It forms heptameric hydrophilic pores that allow the entry of ions, resulting in increased permeability. This process leads to focal, multifocal, or coalescent necrosis of enterocytes and, in severe cases, coagulative necrosis of the entire superficial mucosa separating the intestinal lamina of birds (Figure 2f) [44].

PFOA is encoded in most disease-associated *C. perfringens* strains, except for strains carrying *cpe* on the chromosome [62]. PFOA interacts with cell membrane cholesterol to form pores [33]. It acts synergistically with CPA toxin to affect leukostasis [63] and promotes the expression of adhesion molecules and platelet-activating factors. These toxin-associated features contribute to developing myonecrosis (gas gangrene), haemorrhagic enteritis in calves, and septicaemia (intravascular haemolysis) in humans, including neonates [64–66]. Most recently, it was described that *pfoA *+ strains were associated with preterm infants, including those with necrotizing enterocolitis, and were shown *in vitro* to cause significantly more intestinal cell damage than *pfoA*- strains [11].

CPB2 is an accessory toxin produced by *C. perfrignens* associated with porcine, equine, and bovine enteritis. While the structure of CPB2 and the identification of residues responsible for its antigenicity and association with the membrane of intestinal epithelial cells are under study [67], CPB2 has been reported as a pore-forming toxin. Its action mechanism involves forming cation-selective channels approximately 1.4 nm in diameter in lipid bilayers, leading to altered ion flux and increased intestinal permeability [34].

## Genetic diversity and host specificity of *C. perfringens* toxins

Understanding the molecular patterns influencing interspecies transmission and host adaptation (Figure 3 and Table 1) is crucial for disease prevention and control.

The amino acid sequence diversity of the CPA toxin from 15 strains of *C. perfringens* suggests variation within the signal sequences (six positions), the N-domain (10 positions), C-domain (three positions), and the N- to C-domain linker peptide (one position), with most of these changes conserved potentially impacting the toxin’s resistance to degradation and biological activity [68]. The limited variability of the CPA toxin enhances its affinity for endothelial cells in the host, destroying the endothelial structure; this disruption in peripheral circulation results in myonecrosis [69].

The study of CPB diversity in isolates of toxinotypes B and C has identified that some isolates of type C have four conserved amino acid changes, resulting in two natural variants of the toxin. The sequence modifications in CPB found in toxinotype C isolates lead to increased toxin sensitivity to trypsin, a higher affinity phenotype for host endothelial cells, and increased cytotoxicity. As a result, the molecular diversity of CPB in toxinotype C may contribute to enterotoxaemia and necrotizing enteritis in hosts deficient in trypsin, such as neonatal animals, individuals with diets rich in trypsin inhibitors, and those with pancreatic dysfunction [70].

The genetic diversity of the ETX toxin still needs to be fully elucidated. It has been described that the N-terminal amino acid sequence of ETX is identical in all strains examined to date [71]. Amino acid mutations within domain II of ETX affect pore characteristics and result in changes in cytotoxicity on renal distal tubule cells, leading to pulpy kidney disease in ruminants [72].

The diversity analysis of the ITX components, Ia and Ib, obtained from E-type strains from a calf and a human in France, reveals a very high identity, above 99%, at the amino acid level [73]. Although both toxins show conserved sequences, studies on iota toxin diversity are limited, and a better understanding of their contribution to enterotoxaemia in lambs, rabbits, and calves is required.

The genetic diversity analysis of the CPE toxin gene located on the chromosome, associated with food poisoning, and on plasmids linked to AAD reveals identical nucleotide sequences with similar cytotoxic activity, suggesting a physiological basis for the genotype-disease relationship [74]. Strains harbouring the CPE toxin on the chromosome exhibit superior heat resistance compared to non-food human gastrointestinal disease strains carrying the plasmid CPE gene. This resistance favours their survival in food and contributes to the development of food poisoning [75].

Analysis of NetB sequence diversity in different avian isolates reveals a high degree of conservation despite the presence of an amino acid variant, A168 T, in some isolates. This variant does not significantly affect the physical properties of the encoded protein and does not differ in cytotoxicity. These genetic characteristics of the NetB toxin provide additional evidence that NetB constitutes an essential virulence factor in the pathogenesis of necrotic enteritis in birds [76].

Although the genetic diversity of the PFOA toxin and its influence on disease development is not well understood, the nucleotide sequence of the *pfoA* gene has been reported to exhibit approximately 86% identity with a membrane-damaging thiol-activated alveolysin detected in *C. perfringens* isolates IQ2 (type E) and IQ3 (BEC-positive) [18]. This toxin also shares a 60% identity with the streptolysin O gene and a 48% identity with the pneumolysin gene [77].

The expression of CPB2 toxin in *C. perfringens* strains varies according to host species, with two variants identified: consensus in porcine strains and atypical in non-porcine species. While the genotype-phenotype correlation is high in the consensus variant, the atypical variant shows only a 50% correlation. Furthermore, the atypical genes in types D and E are more similar to each other than isolates for non-porcine kinds A, B, and C, suggesting divergent evolution and a possible explanation for tropism by the host [78].

A detailed description of other accessory toxins is presented in Table 1 – section B.

## Conclusions

In recent years, efforts have been made to understand how chemical structures underpin the molecular and cellular mechanisms of action of bacterial toxins, including the regions involved in host cell recognition and the receptors that confer specificity to each toxin in different hosts, leading to the development of the pathological state. However, although *C. perfringens* is part of the “resident” microbiota, studies need to probe the genetic diversity of each toxin in healthy animals and humans. This is crucial in understanding whether changes in the toxins render them less toxic or lead to their deactivation, ultimately contributing to a more “commensal” lifestyle.

Future research could focus on additional sampling of isolates from healthy and diseased hosts and ecological niches to unravel broader evolutionary aspects, phylogenetic relationships, and genetic diversity of this bacterium, including a comprehensive examination of toxin-associated gene makeup. Further elucidation of the precise association between toxin-specific molecular and structural changes linked with host affinity will enhance knowledge about the diversity of this important pathogen.

In addition, gut microbiota studies are needed to understand how they may influence resistance to *C. perfringens* colonization, competition for space and specific nutrients, or toxin function.

Understanding the interactions, genetic diversity, precise targets, and selective toxicity mechanism of *C. perfringens* toxins may open new avenues for preventative and therapy development. This could involve the rational design of potent pharmacological inhibitors/compounds and identifying new vaccine targets to counteract the effects of toxins, ultimately improving human and animal health.

## Supplementary Material

Supplementary_1
