# Supplementary figures and images for: Unveiling the pathogenic mechanisms of Clostridium perfringens toxins and virulence factors
Source: Emerg Microbes Infect. 2024 Apr 9;13(1):2341968. doi: 10.1080/22221751.2024.2341968 (PMC11057404; doi:10.1080/22221751.2024.2341968)

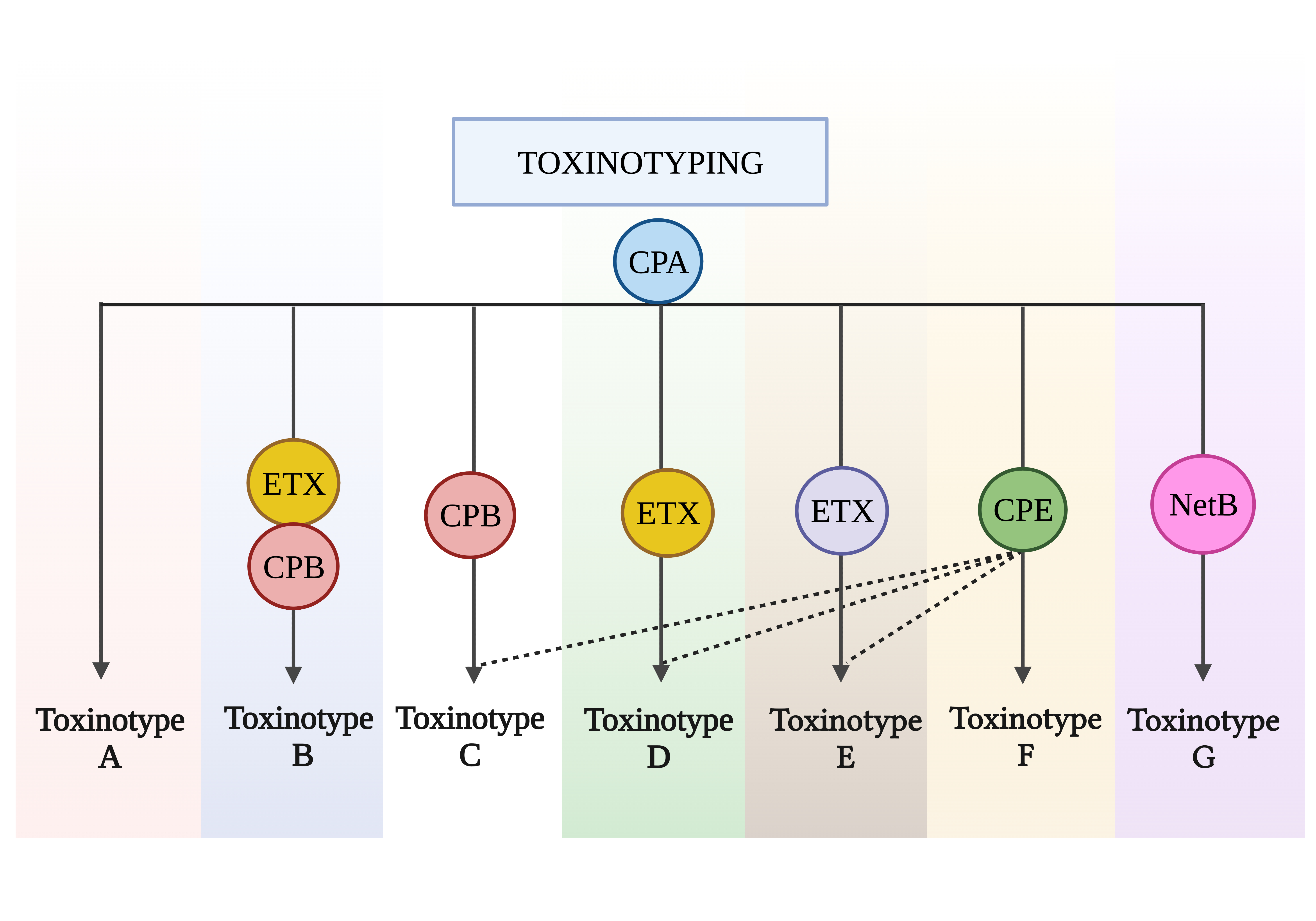

Supplement: Supplementary_1 [file TEMI_A_2341968_SM9894.jpg]
